# DNA Polymerases for Whole Genome Amplification: Considerations and Future Directions

**DOI:** 10.3390/ijms24119331

**Published:** 2023-05-26

**Authors:** Carlos D. Ordóñez, Modesto Redrejo-Rodríguez

**Affiliations:** 1 CIC bioGUNE, Bizkaia Science and Technology Park, Building 800, 48160 Derio, Spain; 2 Department of Biochemistry, Universidad Autónoma de Madrid and Instituto de Investigaciones Biomédicas “Alberto Sols”, CSIC-UAM, 28029 Madrid, Spain

**Keywords:** DNA polymerase, whole genome amplification, PCR, multiple displacement amplification, fidelity, processivity, translesion synthesis, Φ29 DNA polymerase, *Bst* DNA polymerase

## Abstract

In the same way that specialized DNA polymerases (DNAPs) replicate cellular and viral genomes, only a handful of dedicated proteins from various natural origins as well as engineered versions are appropriate for competent exponential amplification of whole genomes and metagenomes (WGA). Different applications have led to the development of diverse protocols, based on various DNAPs. Isothermal WGA is currently widely used due to the high performance of Φ29 DNA polymerase, but PCR-based methods are also available and can provide competent amplification of certain samples. Replication fidelity and processivity must be considered when selecting a suitable enzyme for WGA. However, other properties, such as thermostability, capacity to couple replication, and double helix unwinding, or the ability to maintain DNA replication opposite to damaged bases, are also very relevant for some applications. In this review, we provide an overview of the different properties of DNAPs widely used in WGA and discuss their limitations and future research directions.

## 1. Introduction

For more than 60 years, DNA polymerases (DNAPs) have been one of the cornerstones for the development of molecular biology, genetic engineering, and the current genomic era. Many applications of fundamental importance in modern biotechnology and biomedicine, DNA amplification methods (including polymerase chain reaction (PCR)), and some of the most cutting-edge DNA sequencing technologies would not be possible without advances in the structure and function of DNAPs [1]. Among these techniques, whole genome amplification (WGA) refers to the amplification of genome DNA sequences in a sample that can range from a single virus [2] to polyploid eukaryotic genomes [3] or complex metagenomes [4]. WGA typically starts from a tiny DNA input, providing enough genetic material for subsequent analyses on the order of micrograms.

WGA has been extensively improved and customized since the last decade of the 20th century, and it can be carried out by two main approaches [5,6]. PCR-based WGA methods use repeated heat denaturation cycles to perform amplification. While conventional PCR relies on sequence-specific primers, PCR-based WGA employs primers that either contain complete or partial random sequences or match genome repetitive sequences, allowing the amplification of the entire genome. Alternatively, multiple displacement amplification (MDA) profits of strand-displacement capacity of specific DNAPs perform isothermal WGA. MDA protocols are carried out at a constant temperature and primed by either short random primers or an accessory enzyme with RNA/DNA primase activity (see Section 2.2).

Competent whole (meta)genome amplification aims at reproducing perfectly the entire input genomic sequence. To achieve this, several aspects of the process must be carefully considered in order to achieve a reliable and highly yielded DNA amplification. One of the most critical factors is fidelity to the template sequence (Section 4.3), which requires a high accuracy of the DNAP in forming correct base pairs during the DNA synthesis process. This accuracy is enhanced for replicases endowed with proofreading 3′-5′ exonuclease activity. Enzymes with low fidelity introduce more errors during DNA synthesis, hindering accurate representation of the original DNA sample and increasing the number of false positives in sequence variant calling, especially single-nucleotide variants (SNVs). Another key factor is the ability of the DNAP to perform processive DNA synthesis (Section 4.1), which is the uninterrupted synthesis of long amplicons by a single DNAP molecule. Replication processivity is key to genomic amplification and it can be influenced by several factors, such as replication errors or DNA sequence context (GC content, secondary structures…). Moreover, processivity is closely related to the ability of the DNAP to perform strand displacement (Section 4.2), which allows DNAP to displace the non-complementary strand during DNA synthesis and proceed with processive DNA synthesis.

Evaluation of competent WGA analysis by deep sequence is usually assessed by sequence coverage, which can be measured by two main parameters: depth, i.e., the number of reads containing each nucleotide in the sequence, and breadth, i.e., the proportion of nucleotides in the consensus sequence obtained relative to the length of the original sequence at the depth obtained [7]. A good coverage is required to perform detailed genomic analysis and detect population variants, copy number variations (CNVs), and structural variants (SVs).

In this review, we compare the current WGA techniques, highlighting the key characteristics that make DNA polymerase suitable for these methods (Section 4). Moreover, we focus on recent research on DNAPs to develop novel WGA methods and protocols (Section 6) [8,9].

## 2. Overview of Whole Genome Amplification Methods

### 2.1. WGA Protocols Based on PCR

Unquestionably, polymerase chain reaction was one of the most groundbreaking biotechnological methods developed in the 20th century [10,11]. PCR’s crucial role in detecting pathogens is well known, as it has been widely used to investigate viruses and microorganisms, including SARS-CoV-2, HIV, cytomegalovirus, influenza, *E. coli*, or tuberculosis [12,13]. The specificity of PCR for detecting DNA sequences is supported by specific oligonucleotides that hybridize to the target sequences. However, in the case of WGA, the goal is to fully amplify all DNA molecules of the sample, irrespective of its sequence. In fact, the DNA input sequence is often unknown, and WGA is a prior step required for sequencing and further analysis. In these situations, the high specificity of PCR would be a disadvantage. Likewise, PCR methods can also have some other shortcomings, such as limitations in the amplification of long fragments or sequences with very high GC content [14]. Nonetheless, a great variety of PCR protocols have been successfully developed to overcome this limitation and to achieve competent whole genome amplification.

**Figure 1:**
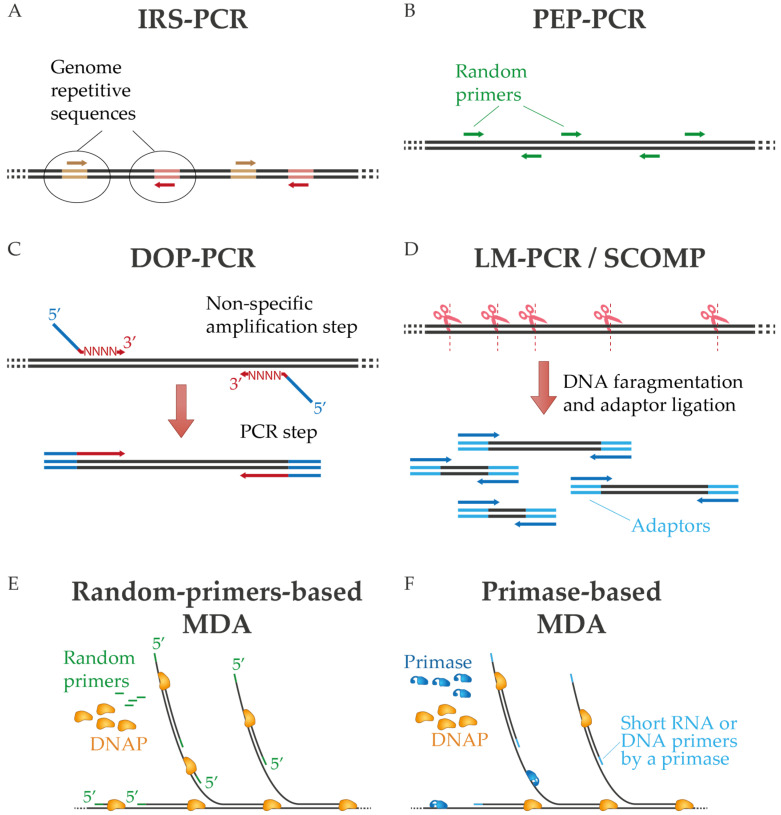
Diagram of diverse PCR-based WGA techniques (**A**–**D**) and multiple displacement amplification (**E**,**F**). In MDA are represented methods that use random primers or a dedicated primase. See text for details.

One of the first approaches of non-specific amplification by PCR was interspersed repetitive sequence PCR (IRS-PCR), in which oligonucleotides are directed to repetitive sequences of the genome [15,16]. The need to know these repetitive sequences in advance limits its use to applications related to known samples, mostly involving the human genome (Figure 1A). Alternatively, sequence-independent, single-primer-amplification (SISPA) was developed by Reyes and Kim [17] to amplify unknown sequences using random primers tagged with a known sequence. SISPA has been mainly used for amplification and detection of metaviromes due to the possibility of generating a sufficient amount of cDNA for cloning and then sequencing [18,19], although this method has been further developed [20,21]. In degenerate oligonucleotide primed PCR (DOP-PCR), partially degenerate oligonucleotides are used to perform primary non-specific amplification followed by exponential replication by PCR. In this technique, the oligonucleotides have random 3′ tails that can anneal throughout the genome during the first rounds of amplification and a fixed 5′ region for PCR amplification during subsequent cycles (Figure 1C) [22,23]. A related approach is primer extension preamplification PCR (PEP-PCR), in which completely random primers are used to generate an amplified representation of the original input that is subsequently further amplified (Figure 1B) [24,25,26]. In ligation-mediated PCR (LM-PCR), on the other hand, the genomic sample is digested by chemical cleavage to generate 5′-phosphate-free ends that are ligated with a linker. This linker provides a common sequence for amplification by PCR [27]. Similarly, in comparative genomic single cell hybridization (SCOMP), adaptors are attached to the enzymatically digested genome [28,29] (Figure 1D).

With these true PCR-based approaches, it is possible to achieve complete amplification of the genome from different nucleic acid samples for applications in biomedical or forensic sciences, as described in other comprehensive reviews [30,31]. However, isothermal protocols are widely used for WGA and can achieve higher coverage breadth and lower false positive rates than PCR-based methods.

### 2.2. WGA Protocols Based on Multiple Displacement Amplification (MDA)

Most of isothermal DNA amplification techniques rely on the use of DNAPs endowed with high processivity and the ability to couple DNA replication and unwinding of the double helix (i.e., strand displacement capacity), such as *Geobacillus stearothermophilus* polymerase I Klenow Large Fragment (*Bst*) and *Bacillus virus Φ29* DNA polymerase (Φ29DNAP). These methods offer several advantages over PCR protocols because they can amplify tiny amounts of DNA and, thanks to strand displacement capacity, are performed at a constant temperature, eliminating the need for specialized thermal cycler equipment.

Diverse isothermal DNA amplification protocols, such as loop-mediated isothermal amplification (LAMP), strand displacement amplification (SDA), or rolling circle amplification (RCA), are highly sensitive methods that are useful in bioanalysis and point-of-care diagnostics [32]. However, like PCR, those protocols require prior knowledge of the sequence of interest to design primers, which on the one hand makes them highly sensitive but, on the other hand, unsuitable for generalized DNA amplification, as is the case with whole genome amplification (WGA).

In contrast, multiple displacement DNA amplification (MDA) is a very powerful isothermal whole genome amplification technique that can amplify very small amounts of circular or linear DNA without the need for primer design. In MDA, the newly synthesized product serves as a template without the need for repeated denaturing cycles, resulting in an exponentially growing DNA network at constant temperature. To achieve efficient and reliable genomic amplification, MDA requires a DNAP with high level of processivity and fidelity. While *Bst* DNA polymerase is used in several isothermal amplification methods due to its robustness, performance, and thermostability, its moderate processivity and fidelity make it less appropriate for MDA. Instead, Φ29DNAP is the most suitable enzyme for amplification of large DNA sequences, spanning plasmids, viral and cellular genomes, and metagenomic samples [9,33,34]. Although the first application of Φ29DNAP in isothermal rolling circle DNA amplification dates back more than 30 years [35], this enzyme is still used in most MDA techniques today [36], and it could be considered a fundamental discovery in the field of nucleic acid amplification.

Standard MDA protocols use random primers, commonly DNA hexamers, that anneal erratically throughout the DNA sample, usually after a previous denaturation step. The phosphodiester link between the last two 3′-terminal nucleotides must be replaced with a phosphorothioate bond to resist degradation by the 3′-5′ exonuclease activity of Φ29DNAP [37]. Since the optimal temperature for Φ29DNAP is 30 °C, WGA by MDA with this enzyme can be completed isothermally in a few hours (2–16 h, depending on the protocol) [36]. These primers are then processively elongated by the enzyme, generating long amplicons that subsequently harbor new primers to start new amplicons many times in succession (Figure 1E), as is well described in other publications [36,37]. However, random primers have been reported to be a potential source of bias and artifacts in MDA (see Section 5) [38,39]. Therefore, alternative MDA methods have been developed to avoid the addition of exogenous primers. That is the case of pWGA, which relies on the synthesis of RNA primers by the primase activity of bacteriophage T7 primase/helicase [40] or the entire T4 replisome [41]. Then, the phage replicative DNAP processively extends these RNA primers, generating long amplicons. These amplification methods generate hybrid molecules that contain short RNA regions, which could hinder sequence libraries’ preparation or sequencing. Similarly, TruePrime uses the DNA primase-polymerase (PrimPol) from *Thermus thermophilus* to generate the short primers, which in that case are DNA primers [42], avoiding the generation of hybrid RNA/DNA structures. Even though PrimPol has low fidelity [43], since DNA primers are extended by the high processive Φ29DNAP, these errors are negligible compared with the entire sequence of produced DNA (Figure 1F). PrimPol-based MDA aims to overcome some of the problems of random primers-based MDA methods, particularly in single-cell amplification protocols. Overall, these primase-based methods result in successful amplification of some samples with reduced artifacts and bias against high-GC sequences for some samples [44,45], but others have pointed out some drawbacks [46]. More recently, a primer-independent B-family DNA polymerase (piPolB) has been used to initiate and extend DNA fragments in the absence of primers. The so-called piMDA protocol combines this method with the efficient extension capability of Φ29DNAP to achieve competent amplification, especially for samples with high-GC content [47,48].

A number of hybrid methods have also been developed, such as multiple Annealing and Looping–Based Amplification Cycles (MALBAC), a quasi-linear isothermal amplification method. MALBAC combines cycles of strand-displacement replication and a subsequent PCR amplification. The heat-denaturing cycles requires the employment of thermoresistant enzymes, and, thus, Φ29DNAP is substituted by other DNAP such as *Bst* [9,49].

## 3. Main DNA Polymerases in WGA

As mentioned earlier, besides an essential role in the maintenance of genetic information in vivo, DNAPs play a key role in DNA amplification methods, both in PCR and in isothermal techniques. Several types of proteins exhibit DNA polymerase activity and have therefore been studied and classified to understand both their diversity, evolution, and biological function, as well as their potential biotechnological applications.

DNAPs have been traditionally grouped into non-homologous families that exhibit a mixture of phylogenetic, structural, and biochemical properties (Table 1). The first families to be identified were the A, B, and C, named for homology to *E. coli* DNA polymerases I, II, and III, respectively [50,51]. The A-family is found in bacteria and eukarya, the B-family is present in all three domains of life as well as their viruses and other genetic mobile elements, and the C-family is specific to bacterial genomes. Later on, a group of heterodimeric archaeal DNAPs, the D-family, was discovered that has no homology to the other families. Most members of these families are considered replicative DNAPs. These replicative enzymes are responsible for processively copying most of the genome with high fidelity due to their high nucleotide selectivity and proofreading activity. Some exceptions can be found in the B-family, such as Pol α and ζ, which exhibit very limited processive replication and are instead specialized for specific functions [52,53].

On the other hand, the X-family includes specialized enzymes involved in DNA repair. This group was later proposed to include eukaryotic polymerase β, which does not have homology with the previously described DNAPs [56]. Afterwards, another group of DNAPs, originally known as the UmuC/DinB/Rev1/Rad30 superfamily, was later renamed as the Y-family [57]. Proteins from families X and Y are distributive DNA polymerases, mainly involved in DNA damage tolerance and repair pathways. They can have significant structural differences that allow the interaction with damaged DNA substrates or, in the case of the members of the Y-family, tolerate formation of non-canonical base pairs during translesion DNA synthesis (TLS, see Section 4.4). Thus, replicative DNA polymerases can be replaced during genome replication by a specialized TLS DNA polymerase that can synthesize opposite to damaged bases to circumvent replication arrest [55].

In addition, other proteins with DNA synthesis capacity have been described, although not always referred as DNA polymerases, as in the case of reverse transcriptases [58,59] or the abovementioned DNA primases-polymerases (PrimPols) from the *archaeoeukaryotic primases* superfamily (AEP) [60,61,62].

Processive enzymes are required for WGA applications (Section 4.1) in order to obtain long DNA fragments to achieve high coverage breadth and homogeneous depth. Moreover, high replication fidelity (Section 4.3) also results in more competent genome amplification. Among all the DNAP families, the A- or B-family replicative enzymes are the most commonly used in DNA amplification methods and WGA because of their ability to accurately replicate long DNA strands. Among the best-known members of the A-family used for these applications are thermoresistant bacterial enzymes, such as *Taq*, the abbreviation for the DNA Polymerase I from *Thermus aquaticus* [63], which is a DNAP classically used in PCR protocols, or the aforementioned *Bst*. These thermophilic bacterial enzymes lack 3′-5′ exonuclease activity, which limits their capacity for generating faithful DNA products. In contrast, B-family proofreading DNAPs, such as *Pfu* and *Pwo* polymerase from the hyperthermophilic archaea *Pyrococcus furiosus* and *Pyrococcus woesei*, respectively [64,65,66], or Φ29DNAP [33,34,35,67], can provide high-fidelity DNA amplification (Table 1).

The viral Φ29DNAP is particularly attractive for WGA because of its high fidelity, processivity, and strand displacement (see Section 4.1, Section 4.2 and Section 4.3). For this reason, new Φ29-like DNAPs or variants of Φ29DNAP have been explored in recent decades to increase the yield of WGA protocols. In the following sections, we compare the properties of these and other enzymes that make them good candidates for WGA. We also describe some strategies to obtain new DNAPs or improve them by protein engineering to increase the yield of current WGA methods.

## 4. Key Features of DNA Polymerase for Accurate WGA

There are already several recent excellent reviews on the wide range of protocols for WGA as well as isothermal DNA amplification methods [8,9,36,68]. Here, we focus on key properties of DNAPs used in those methods, such as processivity, strand displacement, fidelity, TLS, and thermostability, which determine proficiency and limitations of these enzymes in WGA. As detailed below, these properties are critical to the success of nucleic acid amplification techniques.

### 4.1. Processivity of DNA Synthesis

The processivity of a DNAP is the length of DNA strand that can be continuously synthesized in a single hit, i.e., without falling off the substrate. Thus, a DNAP with high processivity would be able to generate long amplicons even at low enzyme concentrations, whereas an enzyme with low processivity, or distributive DNAP, would synthesize shorter DNA segments per reaction. In WGA, where large genomic DNA molecules are the target, high processivity is critical to achieve complete coverage.

Although in vivo processivity factors, such as the β-clamp or the PCNA, can increase the processivity of DNA polymerases during DNA replication, most WGA protocols are based on single enzymes rather than complex replisomes. Some exceptions have been successfully developed, such as the abovementioned pWGA that takes advantage of the bacteriophages T7 or T4 replicative machinery [40,41]. Moreover, single-strand binding proteins (SSBs) can also enhance the processivity of DNAPs [69,70]. For example, *Thermus thermophilus* SSB has been employed to increase the efficiency of WGA protocols [71,72]. Nonetheless, generalized use and reproducibility of DNA amplification techniques is easier to achieve with DNAPs with intrinsic high processivity rather than adding additional components to the reaction mixture.

Unlike PCR-based WGA protocols, where the length of the amplicon is determined by the primer pair, in MDA, the length of the amplicon is expected to reach the length of the template, i.e., the whole chromosome (Figure 1). Therefore, highly processive enzymes such as Φ29DNAP or *Bst* are used for this method [3,8]. Among them, Φ29DNAP is even more processive than *Bst*, with the ability to generate ultralong DNA fragments larger than 40 kb [33,35,73]. In addition, other viral enzymes structurally related to Φ29DNAP can be also used for MDA, such as those from bacteriophages Nf and Bam35 (Table 2) [74,75].

### 4.2. Strand Displacement Capacity

Another difference between isothermal amplification and PCR is that in MDA the DNAP would encounter a complementary DNA strand. Thus, similar to in vivo genome replication, processive DNA synthesis during MDA requires the double helix unwinding. In the pWGA method (Section 2.2 above), the bifunctional protein primase/helicase of bacteriophage T7 (gp4) or helicase from T4 (gp41) unzips the non-template strand to facilitate the progress of DNA polymerase as it occurs in vivo [40,41]. Alternatively, MDA can be performed by DNAPs with an intrinsic helicase-like activity, known as strand displacement capacity. Contrary to a standard DNA helicase activity, which couples ATP hydrolysis with helix unwinding, DNA polymerases with strand displacement capacity can open the double helix during processive DNA synthesis [76,77].

**Table 2:** Summary of some characteristics of the most common DNAPs employed in WGA methods. Translesion DNA synthesis (TLS) activity is indicated as the blocking damaged bases that DNAP can synthesize opposite to, corresponding to tetrahydrofuran (THF) (an abasic site analog), thymine glycol (Tg), and thymine dymer (T:T). ND: no data available.

DNA Polymerase	Family	Processivity	5′-3′ Exo	3′-5′ Exo	Strand Displacement	Average Error Rate	TLS	Optimum Temp. (°C)
*Taq*	A	20–50 nt [78,79]	+/− ^c^	−	−	1–2 × 10^−4^	THF [80]	70–75
*Pwo*	B	<*Taq* [81] ^a^	−	+	−	2.4 × 10^−6^	ND	72 ^b^
*Bst*	A	<Φ29DNAP [73] ^a^	+/− ^c^	−	+	1.5 × 10^−5^	ND	65
Φ29DNAP	B	>40 Kb [33,35,73]	−	+	+	5 × 10^−6^	[74]	30
B35DNAP	B	>23 Kb [74]	−	+	+	5 × 10^−6^	THF, Tg, T:T [74,82]	37

^a^ Only approximate comparative references for processivity are available. ^b^ Data has been extracted from the temperature use in diverse scientific publications and commercial protocols [83,84]. ^c^ 5′-3′ exonuclease activity present in wild-type but absent in the commonly used Klenow large fragment.

A-family DNAPs often couple strand displacement to an intrinsic 5′-3′ exonuclease activity, but that would be detrimental for DNA amplification and, thus, A-family DNAPs used in diverse DNA amplification methods are modified to remove the 5′-3′ exonuclease capacity, such as the Klenow large fragment of *E. coli* DNA Pol I [85]. Klenow fragment displays certain strand displacement capacity that can be enhanced in the absence of 3′-5′ exonuclease proofreading activity, which allowed the development of one of the earliest isothermal DNA amplification, named strand displacement amplification (SDA) [86]. Indeed, *Bst* DNAP is actually used generally as a Klenow-like variant without 5′-3′ exonuclease activity in MDA [87]. This DNAP can unroll the non-template strand efficiently, favored by the natural lack of proofreading activity and the high reaction temperature at 65 °C, enabling processive synthesis through double strand DNA (Table 2) [88].

**Figure 2:**
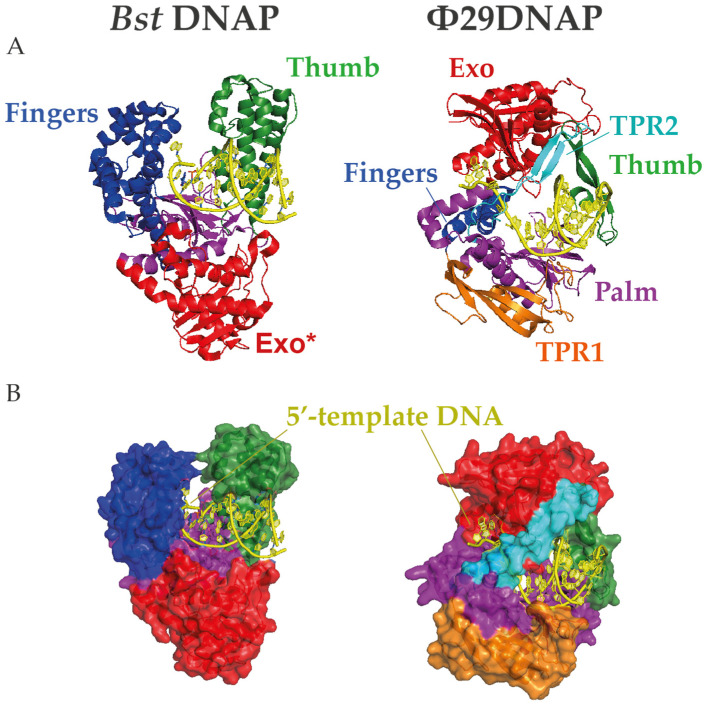
Structures of *Bst* and Φ29 DNA polymerases (DNAPs). (**A**) Cartoon and (**B**) surface representations of the protein structures complexed with DNA in which N-terminal exonuclease domain is highlighted in red and the C-terminal polymerization subdomains palm, finger, and thumb are colored in magenta, blue, and green, respectively. Moreover, the Φ29-specific insertions TPR1 and TPR2 are represented in orange and cyan, respectively. Note that the TPR2 motif encircles the downstream template DNA in a narrow gap, providing a unique mechanism for strand displacement. The crystal structures were obtained from the Protein Data Bank (7K5Q for *Bst* and 2PYJ for Φ29DNAP) and rendered with PyMOL Molecular Graphic System (Schrödinger, LLC).

In contrast, Φ29DNAP can couple processive proofreading DNA synthesis with a competent strand displacement capacity at 30 °C, making it a remarkable exception among monomeric replicative DNA polymerases [89]. Φ29DNAP has a specific insertion, called TPR2, which forms a donut-like shape together with the palm and thumb subdomains that surrounds the downstream DNA and stabilizes the protein-DNA interaction [77]. This ring is too tight to enclose the dsDNA, which favors the separation of the complementary strand in the course of DNA polymerization [90]. Remarkably, the interaction between TRP2 and the thumb to form a ring shape shows sufficient flexibility to allow an appropriate balance between the polymerization and exonuclease activities and even to bind and replicate ssDNA circles (Figure 2) [91]. The amino acid sequence of the TRP2 motif is not well conserved beyond Φ29-related bacteriophages, but this structure can be predicted in other B-family DNAPs and has been shown to be required for processivity and strand displacement in Bam35 DNA polymerase (B35DNAP), a distant viral DNAP with no sequence similarity in the TPR2 motif [74].

### 4.3. Fidelity and Accuracy in DNA Replication and Amplification

Whole genome amplification DNAPs must amplify the entire (meta)genome faithfully and with even coverage. As mentioned above, replicases, enzymes that copy genetic material, typically exhibit high fidelity that makes them suitable for WGA. Fidelity refers to the ability of a DNAP to accurately incorporate the correct nucleotide opposite the template base. The fidelity of a DNAP can be quantified by its error rate (ER), which measures the number of incorrect nucleotides incorporated per unit of nucleotides polymerized. The error rate for each base substitution as well as the insertion or deletion (indel) can be different, and it also depends on the sequence context [74,92,93]. To ensure the preservation of genetic information, DNA polymerases exhibit a broad spectrum of fidelity levels that are often tightly regulated. There are several mechanisms by which DNAPs achieve high fidelity, including: (i) a narrow catalytic pocket that only can accommodate canonical Watson-Crick (WC) base pairs [94]; (ii) an induced-fit mechanism or conformational selection, which allows the fingers subdomain to switch between open and closed conformations when the catalytic site is engaged by a complementary base pair [55]; and (iii) the 3′-5′ exonuclease activity, also known as proofreading, which reduces the error rate (ER) by 10 to 100 times [95,96]. If a mismatch is detected during the DNA replication, the incorrectly synthesized nucleotides can be removed by the exonuclease catalytic center of the proofreading DNAP [97].

Some of the most robust enzymes employed in DNA amplification techniques lack 3′-5′ exonuclease activity (Table 1) [88,98]. That is the case of A-family DNAPs such as *Taq* (ER = 1–2 × 10^−4^) or *Bst* (ER = 1.5 × 10^−5^), which show lower fidelity than competent proofreading enzymes [99,100]. On the other hand, the proofreading DNAP from bacteriophage T7 (ER = 3–15 × 10^−6^) and thermotolerant proteins with 3′-5′ exonuclease such as B-family *Vent* (ER = 3–6 × 10^−5^) or *Pwo* (ER = 2.4 × 10^−6^) have a lower error rate [93,101]. In addition, the replicative enzyme from bacteriophages Φ29 or Bam35, with high fidelity (ER = ~5 × 10^−6^) and other interesting properties, makes them a highly appropriate option for WGA (Table 2) [74,102].

In contrast, Y-family DNAPs possess a solvent-exposed catalytic pocket that maintains loose interactions with the template strand, allowing DNA synthesis even in the presence of bulky damaged bases (Section 4.4). This makes them an ideal resource for DNA damage tolerance, but the absence of a conformational selection mechanism and proofreading capacity result in a relatively high rate of misinsertion during DNA replication [55,94,103]. For this reason, very few members of the Y-family are employed in biotechnological DNA amplification. One enzyme that is sometimes used in various nucleic acid amplification tools is the DNA Pol IV from *Sulfolobus solfataricus* (Dpo4, ER = 6 × 10^−3^) [94,104]. However, due to its low fidelity and processivity, Dpo4 is only applied in blend with *Taq* polymerase for the recovery and of lesion-containing DNA samples or in mutagenic PCR protocols [105,106].

In addition to the intrinsic characteristics of DNA polymerases that determine their fidelity, the metal ion used as a cofactor can also affect the accuracy of DNA replicaton. High-fidelity DNAPs prefer to use magnesium ions (Mg^2+^) as a cofactor [107]. However, DNAPs involved in translesion DNA synthesis, such as some Y-family DNAPs [108] and PrimPol [109], have been reported to naturally use manganese ions (Mn^2+^). Mn^2+^ is a more polarizable ion, and it binds more tightly to the triphosphate moiety. This leads to a reduction in the K_m_ for nucleotides, which in turn increases DNA polymerization capacity, ensuring efficient bypass of DNA lesions [110,111,112]. Furthermore, the variation of metal cofactor can also hinder the proficiency of the in vitro DNA synthesis and thus amplification efficiency. Thus, the use of Mn^2+^ in DNA amplification can reduce the fidelity even of the most faithful DNAPs [102,113]. Therefore, in the context of WGA techniques, where the ultimate goal is to obtain as accurate a full genome amplification as possible, Mg^2+^ is the most commonly use ion in the reactions [24,25,28,38,40,42,114].

### 4.4. Damage Bypass and Translesion DNA Synthesis

Translesion DNA synthesis (TLS) is a DNA damage tolerance mechanism performed by certain DNA polymerases to bypass abnormal or modified template nucleotides. While most replicative DNA polymerases are halted when a lesion is encountered in the template DNA strand, specialized TLS polymerases, mainly Y-family enzymes but also some B-family members, can continue replication by either inserting a nucleotide opposite to the lesion, which is typically the case for abasic sites or small base modifications, or by skipping the damaged nucleotide if they contain larger modifications, such as UV damage or bulky alkylations, resulting in frameshift mutagenesis. However, as mentioned, some specialized TLS DNA polymerases also exhibit decreased selectivity for the correct nucleotide, resulting in very low replication fidelity.

During the last decade, a TLS capacity of diverse replicases has been reported, spanning viral and cellular DNAPs from families A and B [115,116,117,118], which open the possibility of WGA of samples containing damaged bases or modified nucleotides. Among them, the aforementioned B35DNAP exhibits TLS activity in the presence of Mg^2+^ [74] (Table 2). Further, an engineered variant of this DNAP has also been shown to have increased ability to perform processive DNA amplification on damaged templates [119]. On the other hand, fine-tuning the metal cofactors, by exchanging Mg^2+^ by Co^2+^ or by a combination of Mg^2+^ and Mn^2+^, has been reported to enhance the TLS capacity of Φ29DNAP and B35DNAP without significatively decreasing their fidelity [119,120]. Therefore, we envision further developments on this line, by means of the use of tailored enzymes and different metal cofactor combinations that can permit solving the paradoxical contradiction between high fidelity and base damage tolerance, and thus enable the WGA of damaged DNA samples by modulating TLS capacity of high fidelity DNAPs.

### 4.5. Thermoresistance

The yield of many enzymatic reactions can be enhanced by increasing the reaction temperature. Furthermore, high temperature causes the double-strand DNA (dsDNA) denaturalization, increasing the accessibility of the DNAP for challenging regions such as high-GC sequences and, consequently, reducing bias. Hence, thermoresistant proteins have been used in many biotechnological applications including DNA amplification methods. Tolerance to high temperatures is an essential feature for DNAPs in PCR protocols [63]. Therein, the heat-denaturing cycles would inactivate non-thermoresistant enzymes. For this reason, DNAPs employed in PCR-based WGA have classically been found in thermophilic organisms, as *Taq* which is stable at 95 °C or *Pwo*, shows activity even after long treatments at 98 °C [63,83,121].

Contrary to PCR, isothermal DNA amplification occurs at a constant temperature. Due to the lack of heat cycles for DNA denaturing, proteins with great processivity and capacity to unzip the dsDNA are needed (see Section 4.1 above). Among them, pWGA relies on the use of proteins from the replisome of bacteriophage T7 or T4 at 37 °C [40,41,44]. Φ29DNAP optimal temperature is 30 °C and, therefore, the Φ29-based MDA protocols are carried out at this temperature [33,35,42]. The working temperature of Φ29DNAP has been reported as a possible source of bias and artifacts [39,122]. Thus, obtaining protein variants with increased thermostability and thermoresistance have been a longtime goal and several engineered proteins capable of DNA amplification above 40 °C have been reported (see Section 6) [123,124,125,126], which also requires the use of longer random primers (7–10 mer). On the contrary, one of the main interesting features of *Bst* DNAP is its activity at high temperatures (65 °C) (Table 2) [88], which have also been recently successfully increased [127].

In essence, high thermoresistance is crucial for DNA polymerases used in PCR-based WGA protocols, and while not essential, it can be advantageous for MDA-based methods. However, because MDA requires greater processivity and strand displacement capacity, finding suitable DNAPs for these methods is more challenging. As a result, there is a limited selection of DNAPs that can function effectively at the high temperatures required for isothermal WGA.

## 5. The Impact of DNA Polymerases on Limitations and Weaknesses of WGA

In the case of cellular genomes, competent and complete replication of the generic information is highly efficient but also challenging, and many regulatory processes and enzymatic pathways are involved in this task. WGA aims to achieve exponential amplification of a limited amount of genetic material in an unbiased and faithful manner using a single or very few proteins. This gives us an idea of the existence of certain limitations and shortcomings of WGA techniques (reviewed by Sabina and Leamon [39]). These can be roughly reduced to two main categories: sequence bias and artifacts.

Biased amplification led to overlooked sequences, implying allelic unbalance or reduced diversity and missing strains/species in metagenomics studies. The main source of sequence bias is the GC sequence context. High-GC sequences can impair DNA polymerase processivity, especially in isothermal MDA, which may also contribute to the bias toward moderate GC content sequences in Φ29DNAP-based MDA. Random primers have a total GC content of 50%, which could also increase the bias toward extreme GC sequences. Some studies reported increased bias in single-cell MDA protocols [39,128], but a recent work has highlighted differences between kits that may reflect different Φ29DNAP enzyme concentrations [68].

It should be noted, however, that analysis of WGA performance, usually by high-throughput sequencing of unamplified samples and amplified products, reveals deficiencies of all the elements in the procedure, including DNA polymerases as well as other involved factors. As mentioned earlier, the use of oligonucleotide primers can be a source of both types of deficiencies. First, primer binding efficiency varies with different sequences, GC content, and secondary structure of the amplicon. In addition, the presence of random sequences in the primers can lead to artifactual amplification, sometimes referred to as template-independent DNA amplification (TIDA) [129]. Undesired DNA amplification, especially in samples with small amounts of DNA, can also be the result of contaminated DNA or ab initio DNA synthesis leading to primer- and template-independent DNA amplification [130,131]. While the removal of contaminated DNA associated with recombinant DNA polymerases has been studied by several laboratories [132,133,134], ab initio DNA synthesis seems almost disregarded in the WGA literature. This controversial activity has been reported for several thermophilic DNA polymerases and it seems highly stimulated by nuclease activities that increase the available 3′-OH ends [135,136,137,138], although suboptimal temperature, reducing agents, and salt can enhance this activity [139,140]. More recently, we have reported a high abundance of spurious DNA products in MDA reactions by piPolB. This competent creative DNA synthesis is reduced by prior alkaline DNA denaturation, confirming that limited access to the DNA substrate favors ab initio DNA synthesis, and is negligible when piPolB is combined with Φ29DNAP, highlighting the need for high processivity in WGA [47].

Overall, Φ29DNAP-based WGA results in higher amplification yield and lower bias than PCR-based methods [141]. Recent comparative studies of single-cell amplification methods show that RepliG commercial protocol, based on Φ29DNAP and random primers, has the lowest error rate [142] and false-negative single nucleotide variant (SNV) [68]. However, given the exponential progression of MDA, uneven primer annealing at early stages would lead to a strong bias, resulting in lower allelic balance. In addition, the generation of chimeric DNA sequences can be a major drawback of MDA in certain samples [68,122], although experimental conditions can be adjusted to reduce these artifacts.

PCR-WGA protocols are associated with a higher error rate overall, especially those based on non-proofreading DNA polymerases. This results in poorer assemblies and a higher number of false-negative SNV in the amplification of single-cell genomes compared with MDA. Extreme coverage peaks in regions of low-complexity that are not due to primer sequences have also been reported for SISPA [45]. However, PicoFlex and MALBAC can achieve linear amplification and lower allelic bias in single-cell amplification [68]. Interestingly, new LM-PCR techniques using proofreading thermostable DNA polymerases (e.g., Ampli1) show higher coverage [142], and although they have a higher error rate than Φ29DNAP-based MDA, they achieve less allelic imbalance and dropout and fewer chimeric amplicons [68].

## 6. Improvement of WGA by DNA Polymerases Engineering

Given the special features of DNAPs suitable for WGA and the abovementioned limitations of currently available enzymes, the search for new DNAPs with improved characteristics that benefit genomics and biotechnology is an extremely active task. The search for novel, previously unforeseen DNAPs has been very fruitful over the past decade [48,143,144,145], but engineering of known enzymes is a very useful alternative that allows the enhancement of the activity of proteins and confers new properties to them [146].

For example, several DNAPs with improved processivity have already been engineered to replicate longer amplicons [79,147,148,149,150]. In addition, nucleotide selectivity can be increased to obtain more accurate DNA products [147,151]. Some enzymes have been made more robust by conferring tolerance to various replication hindrances, including high salt concentration [150,152], contaminants such as those found in forensic samples [153], or difficult templates such as rich-GC sequences [154]. To develop competitive WGA tools, it is necessary to increase sensitivity to amplify from lower input [155] as well as increasing thermostability [123,124,125,126,127,156]. Furthermore, catalytic activities can be added or modified to change the application of the DNAP, as seen in the *Taq* DNAP variant where proofreading activity was incorporated [157].

Several strategies can be used to improve the above properties. We can divide these approaches into (i) targeted mutagenesis, (ii) directed evolution, and (iii) design of chimeric enzymes. Each of these procedures has been applied to the field of DNAPs, and some of them can be combined to achieve a synergistic effect.

### 6.1. Targeted Mutagenesis

Modification of enzymes can be based on previous knowledge of the structure-function of the protein of interest or related proteins. Despite being a classical approach, it remains widely used today, as exemplified by the defective 3′-5′ exonuclease activity Φ29DNAP reported in 1989 [96], which was recently used to replicate DNA with xenobiotic nucleic acids, i.e., nucleic acid analogues with alternative backbone chemistry [158]. Similarly, various other properties such as replication yield or replication fidelity can be altered [151,159].

Various strategies can be used to select which residues need to be altered to achieve the desired effect in rational design. For example, the processivity of some DNAPs has been enhanced by targeted mutagenesis based on the study of naturally occurring mutations, as in the case of the T4 DNAP [160], or substitution of relevant residues identified by sequence alignments, as in *Pfu* [161]. Computational biology or machine learning approaches can also be used to predict more stable variants, as was done in *Bst* and other DNAPs [127,162].

### 6.2. Directed Evolution

Directed evolution methods have the advantage that they do not require extensive prior knowledge of the structure or sequence of the protein of interest. These methods involve two main steps: (i) generating a library of the enzyme gene to ensure some diversity, and (ii) performing a screen or selection of the most suitable candidate [163].

DNA polymerase sequence randomization can be performed over the entire gene or focused on specific regions by semi-rational design [164,165]. This process can be performed by chemical mutagenesis [166], degenerated oligonucleotides [164], or error-prone PCR [167,168]. Furthermore, other methods can be used to increase diversity, such as molecular breeding, which mimics recombination between genes [153,169] or random fragmentation and reassembly in DNA shuffling [170,171], which are very appropriate strategies for producing chimeric proteins. 

The next step is to select the improved variants. Some general procedures for protein evolution, such as phage display, can also be applied to replicative DNAPs [172,173]. However, because DNAPs are naturally capable of replicating DNA, they can play a leading role in the evolutionary process by performing their own replication. For example, in compartmentalization self-replication (CSR), a DNAP is emulsified and performs auto-replication through multiple rounds of pressure [123,171]. Similarly, droplets containing DNAPs can be selected based on the activity of the protein in response to the fluorescence signal, as described in droplet-based optical polymerase sorting (DrOPS) [174].

Directed evolution has also been successfully employed in some of the DNAPs used for WGA methods, such as *Taq*, *Bst*, or Φ29DNAP [123,169,171].

### 6.3. Fusion or Chimeric Enzymes

Chimeric proteins are composed of sequence fragments or domains from two or more proteins. DNAPs can be created through domain exchange or by actual protein fusion. Domain exchange involves replacing specific domains with the homologous region of another protein, e.g., replacing the defective 3′-5′ exonuclease domain of *Taq* with the homologous domain of Pol I from *E. coli*, resulting in a functional proofreading activity [157], among others [105,147,150,155,175]. Conversely, fusion proteins involve the addition of an extra domain to the parental enzyme. This is a widely successful strategy in DNAPs, especially by fusing DNA-binding domains that increase processivity and salt tolerance in multiple enzymes [150,152,154,156,162] or other characteristics as thermostability [176]. This approach has also been applied to certain DNAPs used for PCR, which have been fused to chromatin-like Sso7d protein, such as *Pyrococcus furiosus* DNAP (*Pfu*) or *Taq* (commercialized as Phusion^TM^ and Sso7D fusion polymerase) [79,177,178,179]. Additionally, enzymes such as *Bst* or *Bst*-like [180,181] and Φ29 DNAPs (QualiPhi^TM^) [148,182] enabled the improvement of these enzymes and made them more efficient and sensitive to isothermal amplification techniques.

In short, protein engineering approaches such as directed evolution, rational design, and chimeric enzymes have shown promise in modifying DNA polymerases to improve their performance in WGA protocols and other applications. Overall, the role of DNA polymerases in WGA techniques is crucial, and understanding their properties and limitations is essential for successful WGA applications.

## 7. Concluding Remarks

Recent improvements in high-throughput sequencing methods and their broad accessibility have reduced the need for routine WGA of genomic and metagenomic samples. However, the original biomass can often be low and valuable, making amplification unavoidable. This is the case for forensic DNA, samples with high amount of contaminant components, fossil genomes, single-cell (meta)genomics, preimplantation genetic testing, or liquid biopsies, among others.

Currently available WGA methods are mostly based on two different approaches, PCR and isothermal MDA. PCR-based WGA offers a variety of approaches based on different designs of oligonucleotide primers that ensure early amplification cycles by nonspecific amplification, favored by random sequences, or by direct ligation of primers. These methods provide good coverage and accurate allelic equilibrium using thermophilic proteins such as *Taq* DNA polymerase. Recently, some proofreading DNA polymerases have been introduced at PCR-WGA, which also provide good accuracy and a low false positive rate for SNVs. However, MDA-based WGA results in high DNA yield with higher coverage and amplification fidelity. Isothermal MDA used for WGA is mostly based on Φ29DNAP and derivatives [36,183].

Φ29DNAP is an outstanding replicase that combines extremely high processivity with strand displacement and proofreading capacities, a set of features that are part of the definition of competent WGA methods. However, it has some drawbacks, such as the need for a pre-existing 3′-OH end to start the amplification reaction, which can be an additional source of artifacts and distortions. The optimal reaction temperature of 30 °C also makes it less suitable for the amplification of high GC sequences. In addition, the stringent selection of correct base pairs and strong proofreading capacity prevents replication of DNA with base damage, making amplification of certain samples more difficult. Recent MDA protocols based on novel and engineered enzymes and the blend of Φ29DNAP with accessory proteins with DNA primase capacity have attempted to alleviate these limitations [42,47,119,123,148], but further innovative research is needed to develop faithful, processive, and flexible DNAPs for new and more competent WGA methods.

## Figures and Tables

**Table 1:** Classification of DNA families and examples of the best-known members from different organisms [52,53,54,55]. Underlying names indicate DNAPs employed in MDA and WGA techniques. Abbreviations: *Bst*: *Geobacillus stearothermophilu*, *Pwo*: *Pyrococcus woesei*, *Taq*: *Thermus aquaticus*, ND: non-detected.

DNAP Family	Bacteria	Archaea	Eukarya	Virus
**A**	Pol I [*Bst*, *Taq*]	ND	Pol γ	T7
**B**	Pol II	Pol B1, B2, B3 [*Pwo*]	Pol α, δ, ε, ζ	Φ29, Bam35, HSV, RB69
**C**	Pol III	ND	ND	ND
**D**	ND	Pol D	ND	ND
**X**	Pol X	ND	Pol β, λ, μ	ASFV
**Y**	Pol IV (DinB), Pol V (UmuCD)	Dpo4, Dbh	Pol η, ι, κ, Rev1	ND

## Data Availability

No additional data available.
